# Lancisi sign: giant C-V waves of tricuspid regurgitation

**DOI:** 10.1007/s11739-015-1384-4

**Published:** 2016-01-13

**Authors:** Bartosz Hudzik, Lech Poloński, Mariusz Gąsior

**Affiliations:** Third Department of Cardiology, Silesian Centre for Heart Disease, SMDZ in Zabrze, Medical University of Silesia in Katowice, Curie-Sklodowska 9, 41-800 Zabrze, Poland

A 69-year-old woman with a history of surgical mitral valve repair for severe mitral regurgitation 12 years prior presented with a 6-month history of dyspnea on exertion (NYHA functional class II), exercise intolerance, and swelling of her abdomen and lower limbs. On jugular venous examination, giant systolic pulsations with prominent V-waves, known as the *Lancisi* *sign* or C-V waves, were noted (Fig. 1a, Video appendix 1 and 2). On auscultation, a loud first heart sound was audible, with a loud pulmonary component of the second heart sound along with an apical mid-diastolic rumble. A holosystolic murmur at the left lower sternal border that increased during inspiration was also noted. Lower limb edema, ascites and an enlarged, pulsatile liver were present. Transthoracic echocardiography demonstrated normal left ventricular function with an ejection fraction of 55 % and a normal function of the mitral valve. It also confirmed the presence of a dilated right atrium and right ventricle and a severe tricuspid regurgitation (TR) with a moderately reduced right ventricular function. The patient was referred for a surgical tricuspid annuloplasty, but given an increased operative mortality risk refused to undergo the operation.

**Fig. 1:**
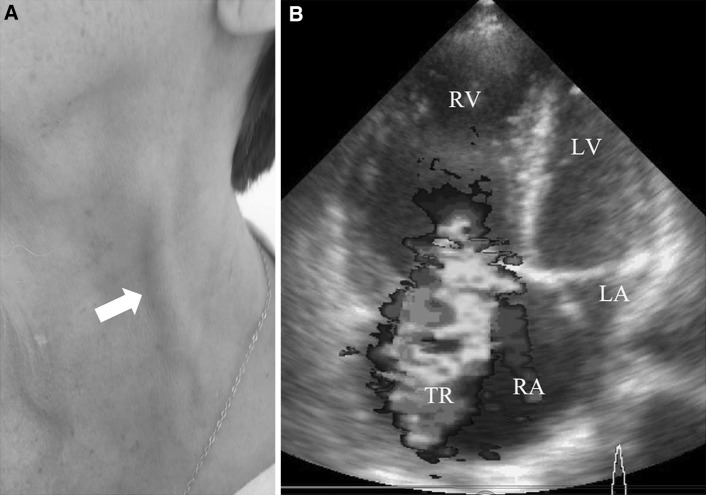
**a** Giant systolic pulsations with prominent V-waves, known as the *Lancisi* *sign* or C-V waves (please see on-line Video 1 and 2); **b** transthoracic echocardiography 4-chamber view demonstrating a dilatated right atrium and right ventricle and severe tricuspid regurgitation with a moderately reduced right ventricular function (*RA* right atrium, *RV* right ventricle, *TR* tricuspid regurgitation, *LA* felt atrium, *LV* left ventricle)

TR is a relatively common abnormality. Since this condition is frequently asymptomatic and may not be detected on routine physical examination, it is often diagnosed solely by echocardiography [1]. Usually, there are no specific signs or symptoms. Yet, in case of severe TR, giant C-V waves or the *Lancisi sign* can be found on the jugular venous examination [1]. With increasing tricuspid regurgitation, there is an increased backflow of blood to the right atrium during systole. In patients with severe tricuspid regurgitation, the V wave of tricuspid regurgitation merges with the C wave forming a single prominent C-V wave that is often mistaken for the large carotid-pulse wave of severe aortic regurgitation. Other signs and symptoms include painful hepatosplenomegaly, ascites, and peripheral edema [2]. Auscultation reveals a loud first heart sound with a loud pulmonary component of the second heart sound, an apical mid-diastolic rumble, and a holosystolic murmur at the left lower sternal border. Generally, tricuspid valve disease occurs secondary to left-sided heart valve disease, in particular mitral valve disease (i.e., functional TR) (Appendix 3). It is a marker of adverse outcome, and patients with moderate/severe TR have a worse prognosis [3, 4]. Primary TR (Appendix 3) is treated surgically if severe, and the patient is symptomatic. However, during concomitant left-sided heart valve surgery, a moderate/severe secondary TR with either raised pulmonary artery pressures or tricuspid annular dilatation should also be treated [3, 4].

## Electronic supplementary material

Below is the link to the electronic supplementary material.

Video appendix 1. Giant systolic pulsations with prominent V-waves, known as the *Lancisi* *sign* or C-V waves seen on jugular venous examination – a distinctive feature of tricuspid regurgitation. (MOV 6689 kb)

Video appendix 2. Giant systolic pulsations with prominent V-waves, known as the *Lancisi* *sign* or C-V waves seen on 006Augular venous examination – a distinctive feature of tricuspid regurgitation. (MOV 9541 kb)

Appendix 3. Primary and secondary causes of tricuspid regurgitation. (DOCX 14 kb)
