# Supplementary material for: Lancisi sign: giant C-V waves of tricuspid regurgitation
Source: Intern Emerg Med. 2016 Jan 13;11(8):1139–40. doi: 10.1007/s11739-015-1384-4 (PMC5114320; doi:10.1007/s11739-015-1384-4)
Supplement: Supplementary file 3 — Appendix 3. Primary and secondary causes of tricuspid regurgitation. (DOCX 14 kb) [file 11739_2015_1384_MOESM3_ESM.docx]

| Primary tricuspid regurgitation | Secondary tricuspid regurgitation |
| --- | --- |
| - Chest trauma – blunt or penetrating injuries - Infective endocarditis - Ebstein's anomaly, the most common form of congenital disease affecting the tricuspid valve - Rheumatic fever - Carcinoid heart disease - Iatrogenic – pacemaker lead trauma - Myxomatous degeneration associated with tricuspid valve prolapse, which occurs in as many as 40 percent of patients with prolapse of the mitral valve - Tumors (e.g., myxoma) - Connective tissue disorder (e.g., Marfan syndrome) - Marantic endocarditis in systemic lupus erythematosus or rheumatoid arthritis - Toxic (eg, Phen-Fen valvulopathy or methysergide valvulopathy) | - Left-sided heart failure - Mitral stenosis or regurgitation - Aortic stenosis or regurgitation - Primary pulmonary disease — cor pulmonale, pulmonary embolism, pulmonary hypertension of any cause - Left to right shunt — atrial septal defect, ventricular septal defect, anomalous pulmonary venous return - Eisenmenger syndrome - Stenosis of the pulmonic valve or pulmonary artery |

Appendix 1. Primary and secondary condition leading to tricuspid regurgitation.
